# Responsiveness of cardiometabolic-related microbiota to diet is influenced by host genetics

**DOI:** 10.1007/s00335-014-9540-0

**Published:** 2014-08-27

**Authors:** Annalouise O’Connor, Pamela M. Quizon, Jody E. Albright, Fred T. Lin, Brian J. Bennett

**Affiliations:** 1 UNC Chapel Hill Nutrition Research Institute, 500 Laureate Way, Kannapolis, NC 28081 USA; 2 Department of Genetics, University of North Carolina Chapel Hill, Chapel Hill, NC 27599 USA; 3 Department of Nutrition, University of North Carolina Chapel Hill, Chapel Hill, NC 27599 USA

## Abstract

Intestinal microbial community structure is driven by host genetics in addition to environmental factors such as diet. In comparison with environmental influences, the effect of host genetics on intestinal microbiota, and how host-driven differences alter host metabolism is unclear. Additionally, the interaction between host genetics and diet, and the impact on the intestinal microbiome and possible down-stream effect on host metabolism is not fully understood, but represents another aspects of inter-individual variation in disease risk. The objectives of this study were to investigate how diet and genetic background shape microbial communities, and how these diet- and genetic-driven microbial differences relate to cardiometabolic phenotypes. To determine these effects, we used the 8 progenitor strains of the collaborative cross/diversity outbred mapping panels (C57BL/6J, A/J, NOD/ShiLtJ, NZO/HILtJ, WSB/EiJ, CAST/EiJ, PWK/PhJ, and 129S1/SvImJ). 16s rRNA profiling of enteric microbial communities in addition to the assessment of phenotypes central to cardiometabolic health was conducted under baseline nutritional conditions and in response to diets varying in atherogenic nutrient (fat, cholesterol, cholic acid) composition. These studies revealed strain-driven differences in enteric microbial communities which were retained with dietary intervention. Diet–strain interactions were seen for a core group of cardiometabolic-related microbial taxa. In conclusion, these studies highlight diet and genetically regulated cardiometabolic-related microbial taxa. Furthermore, we demonstrate the progenitor model is useful for nutrigenomic-based studies and screens seeking to investigate the interaction between genetic background and the phenotypic and microbial response to diet.

**Electronic supplementary material:**

The online version of this article (doi:10.1007/s00335-014-9540-0) contains supplementary material, which is available to authorized users.

## Introduction

The intestinal microbiome is associated with susceptibility to and development of several chronic metabolic diseases including diabetes (Larsen et al. 2010), obesity (Turnbaugh et al. 2008), and cardiovascular disease (Karlsson et al. 2012). Negative changes in host adiposity, metabolic syndrome status, and insulin sensitivity can be directly induced by microbial dysbiosis (Ridaura et al. 2013; Vijay-Kumar et al. 2010; Vrieze et al. 2012). Considering the inter-individual variability at the level of the microbiome (Eckburg et al. 2005; Qin et al. 2010), detailed studies integrating the intestinal microbiome with disease risk complements current genome wide association studies and other efforts seeking to understand heterogeneity in health and disease status. We, among others (Kaput 2008; Perez-Martinez et al. 2013) recognize that a ‘one size fits all’ approach to optimal nutritional and health status is not addressing the current epidemics of obesity, cardiovascular disease, and diabetes. In particular, understanding how microbial diversity and specific microbial species affect clinical phenotypes and risk of disease is necessary. Research models which simultaneously permit both dietary- and genetic-driven perturbations are an essential step in developing personalized approaches to nutrition and medicine.

As the role of genetics in driving disease-susceptibility is being more fully elucidated, the influence of genetic background on the regulation of microbial diversity is also becoming established. Several groups have reported that enteric microbial composition is a heritable trait (Tims et al. 2013; Turnbaugh et al. 2010), although results from twin studies have been discordant (Turnbaugh et al. 2009). However, studies using mouse genetic panels, as well as those exploiting single-gene mutations, have consistently shown an effect of host genetics on intestinal microbial community structure (Benson et al. 2010; Kovacs et al. 2011; McKnite et al. 2012; Parks et al. 2013a; Spor et al. 2011; Toivanen et al. 2001), and may have increased power to detect genotype-driven microbial differences, as murine studies allow for tight control over environmental factors (Benson et al. 2010).

In addition to the influence of genetic background, significant advances have been made in understanding environmental determinants of microbial structure including maternal effects, cage mates, gender, and diet (Spor et al. 2011). As a source of essential nutrients for the intestinal microbiota, host-consumed diet is an important determinant of microbial community structure in the intestine, and dynamic changes in both mouse and human microbial populations occur in response to dietary intervention (Spor et al. 2011). Although environmental influences have a strong effect, and can be dominant in certain cases, genotype–environmental interactions have been shown to contribute to microbial diversity (Parks et al. 2013a), as well as risk of disease (Parks et al. 2013a; Srinivas et al. 2013).

It is not known how host genotype-driven differences in the intestinal microbiome are related to host cardiometabolic phenotype. Using a segregating panel of mice phenotyped for clinically relevant metabolic and atherogenic makers, the objectives of this study were to identify host-genetic-derived differences in the intestinal microbiome and determine how these differences are related to host phenotype under baseline nutritional intake, and following consumption of an atherogenic diet. Recently, a “multiparent advanced generation inter-cross” (MAGIC) population was developed from 8 inbred mouse strains, and is referred to as the diversity outbred (DO) mouse population (Churchill et al. 2012). The DO mice are mosaics of C57BL/6J, A/J, NOD/ShiLtJ, NZO/HILtJ, WSB/EiJ, CAST/EiJ, PWK/PhJ, and 129S1/SvImJ, and these mice complement another large endeavor called the collaborative cross (CC)(Aylor et al. 2011). These eight founder strains of the CC/DO are genetically diverse and capture ~90 % of the known genetic variation in the mouse (Roberts et al. 2007). Here we present data investigating the microbial community diversity in the CC/DO founder stains. Discriminatory microbiota are related to cardiometabolic phenotypes, and the microbial and phenotypic response to dietary factors is investigated and discussed. We demonstrate that this model is useful for nutrigenomic-based studies seeking to investigate the interaction between genetic background, and the phenotypic and microbial response to diet.

## Materials and methods

### Mouse handling

All experiments were approved by the Institutional Animal Care and Use Committee (IACUC) at the North Carolina Research Campus (NCRC). All mice used in these studies were female. Mice were purchased from Jackson Laboratories (Bar Harbor, ME, USA) at 4 weeks of age. Mice were group housed by strain (2 cages/strain, 4 animals/cage) under standard conditions (12 h light:dark, temperature- and humidity-controlled conditions), and received *ad lib* access to water and a nutritionally complete purified synthetic diet containing 9.4 % kcal from fat, 75.9 % kcal from carbohydrate and 14.7 % kcal from protein (AIN-93M; (#D10012M); Research Diets Inc, New Brunswick, NJ, USA). After 4 weeks on AIN-93 M, mice were randomized to diet (*n* = 4/diet group) for a further 16 weeks as follows: (1) a defined atherogenic diet, containing 20 % kcal as fat, 20 % kcal as protein, 40 % kcal from carbohydrate, 1.25 % cholesterol, and 0.5 % cholic acid, (#D12109C, Research Diets Inc, New Brunswick, NJ, USA), abbreviated to high fat cholic acid (HFCA) diet; or, (2) a low-fat cholesterol-containing diet without cholic acid (10 % kcal as fat, 20 % kcal as protein, 70 % kcal as carbohydrate, 1.25 % cholesterol) (#D12104C, Research Diets Inc, New Brunswick, NJ, USA), abbreviated to low-fat (LF) diet. The protein sources (casein and L-cystine) were consistent across diets, as were sources of carbohydrate (cornstarch, maltodextrin, sucrose). Additionally, the type and amount of polysaccharide (cellulose) were comparable between diets. The source of fat (soybean oil for AIN-93M versus soybean oil plus cocoa butter for HFCA and LF) varied between the diets. The increased levels of cholic acid, cholesterol, and dietary fat were selected to induce hyperlipidemia and atherosclerosis (Getz and Reardon 2006). A 16 weeks dietary intervention is sufficient to observe aortic lesion formation (Hyman et al. 1994).

### Body composition

Body composition (proportion of fat mass) was assessed using EchoMRI™-100H (Echo MRI LLC, Houston, TX, USA).

### Plasma metabolic markers

Mice were fasted for 4 h before blood draw via retro-orbital bleed. Blood was collected into EDTA-containing tubes and plasma separated by centrifugation at 10,000×*g* for 10 min. Plasma triacylglycerol (TAG), total cholesterol, and glucose were measured by Biolis 24i Analyzer (Carolina Liquid Chemistries, Winston-Salem, NC). Insulin was quantified using the Alpco Mouse Ultrasensitive Insulin ELISA assay (Alpco, Salem, NH); samples and controls were run in duplicate, and optical densities were measured at 450 nm using a microplate reader and analyzed with Gen5 Data Analysis Software (Bio-Tek, Winooski, VT, USA). Plasma TMAO quantification was performed at the UNC Chapel Hill NORC Choline Metabolite Core using liquid chromatography-stable isotope dilution-multiple reaction monitoring mass spectrometry (LC-SID-MRM/MS). Briefly, plasma was extracted with three volumes of acetonitrile spiked with internal standards TMAO-d9 (DLM-4779-1, Cambridge Isotope Laboratories), incubated on ice for 10 min, and centrifuged at 15,000×*g* for 2 min. Supernatants were then collected for instrumental analysis. Chromatographic separations were performed on an Atlantis Silica HILIC 3 µm 4.6 × 50 mm column (Waters Corp, Milford, USA) using a Waters ACQUITY UPLC system. The column was heated to 40 °C, and the flow rate was maintained at 1 mL/min. The gradient was 5 % A for 0.05 min, to 15 % A in 0.35 min, to 20 % A in 0.6 min, to 30 % A in 1 min, to 45 % A in 0.55 min, to 55 % A in 0.05 min, at 55 % A for 0.9 min, to 5 % A in 0.05 min, at 5 % A for 1.45 min, where A is 10 % acetonitrile/90 % water with 10 mM ammonium formate and 0.125 % formic acid and B is 90 % acetonitrile/10 % water with 10 mM ammonium formate and 0.125 % formic acid. TMAO and its corresponding isotope were monitored on a Waters TQ detector using characteristic precursor-product ion transitions: 76 → 58 for TMAO, 85 → 66 for TMAO-d9. Concentrations of each metabolite in samples were determined from its calibration curve using peak area ratio of the metabolite to its isotope.

### Microbiome Sample Processing

At baseline (on the AIN-93 diet), the mice were singly housed for 24-h prior to fecal collection. For the final 4-h of this period, mice were transferred to fresh bedding, food was removed and feces were collected from each cage at the end of this 24-h period for use in down-stream microbiome studies. After 16 weeks of diet treatment, mice were fasted for 4 h, blood samples collected and mice were euthanized by isoflurane overdose and cecal samples collected. Frozen (−80 °C) fecal samples (1 pellet per animal) or cecal contents (0.5 mg per animal) were homogenized in lysis buffer (TissueLyser LT; Qiagen, Germantown, MD, USA) (50 oscillations/second) for 120 s. DNA was extracted from samples using the Maxwell^®^ 16 Tissue DNA Purification Kit (Promega, Madison, WI) on a Maxwell^®^ 16 Instrument (Promega, Madison, WI, USA). *One Step*™ PCR Inhibitor Removal Kit (Zymo Research Corp., Irvine, CA, USA) was used to remove contaminants which may inhibit downstream PCR. Isolated DNA was stored at −20 °C until further use. Microbial community composition was assessed by 16s rRNA gene sequencing. Briefly, DNA encoding the V4 region of the 16s rRNA gene was amplified using bar-coded fusion primers (F515/R806) (Caporaso et al. 2012). The reverse PCR primer is barcoded with a 12-base error correcting Golay code to facilitate multiplexing, and both PCR primers contain sequencer adapter regions [for full primer details see (Caporaso et al. 2012)]. Briefly, genomic DNA samples (10 ng/reaction) were amplified in triplicate (KAPA HiFi PCR Kit, KAPA BioSystems, Woburn, MA, USA) with 10 µM final primer concentration. Reactions were held at 95 °C for 3 min to denature DNA, followed by 35 amplification cycles of 94 °C for 30 s, 50 °C for 60 s, and 72 °C for 90 s, with a final 10 min extension at 72 °C. Each set of triplicate amplicons were cleaned using the Wizard^®^ SVGel and PCR System (Promega, Madison, WI, USA), and quantified using Qubit dsDNA HS Assay kit (Invitrogen, Oregon, USA). PCR products were run on an Experion™ 1 K DNA Chip (Bio-Rad, Hercules, CA, USA) to assess DNA quality and confirm PCR product size. A composite sample for sequencing was created by combining equimolar ratios of amplicons from the individual samples. This amplicon mixture was sequenced (2 × 250 bp paired end) on a MiSeq^®^ System (Illumina; San Diego, CA, USA).

### 16s ribosomal RNA sequencing-based analysis of intestinal microbial community structure

Raw sequence data were analyzed using Quantitative Insights Into Microbial Ecology (QIIME) (Caporaso et al. 2010b) version 1.8.0. Forward and reverse paired-end reads were stitched with the ea-utils fastq-join program (http://code.google.com/p/ea-utils/) through QIIME. 150 bp was set as the minimum overlap, and the effect of stitching error stringency was assessed by varying the maximum permitted error (0, 1, 3, 5 %). After paired-end stitching, the raw fastq sequence file was demultiplexed in QIIME, wherein each read meeting quality criteria (Phred score >25), was assigned to a sample ID. Sequences were then clustered into operational taxonomic units (OTUs) *de novo* using uclust (Edgar 2010), and a similarity threshold of 97 %. Representative sequences (most abundant sequence in OTUs) were chosen, aligned to GreenGenes Core reference alignment (DeSantis et al. 2006) using PyNAST (Caporaso et al. 2010a). Taxonomic classification was assigned with Ribosomal Database Project (RDP) Classifier 2.2 (Wang et al. 2007) through QIIME, and a phylogenetic tree built using FastTree 2.1.3 (Price et al. 2010). The resulting biom-formatted OTU table was filtered to remove singletons, and rarefied to an even sampling depth of 51,000 reads/sample. Assessments of alpha-diversity (number of observed species, Shannon Diversity Index, GINI co-efficient) were conducted in QIIME. The biom-formatted OTU table, mapping file, and phylogenetic tree were imported into R Studio (v 3.0.1) via Phyloseq (McMurdie and Holmes 2013), and beta-diversity assessed by UniFrac (Lozupone and Knight 2005). Principle Co-Ordinates Analysis (PCoA) was applied to reduce the dimensionality of the resulting distance matrix. To determine the influence of strain and diet on global microbial communities, UniFrac distance matrices were passed to the R package Vegan (Jari Oksanen et al. 2013) for analysis of similarity (ANOSIM), and Permutational Multivariate Analysis of Variance (PERMANOVA). Hierarchical clustering (Euclidean distance) was conducted using phyla level relative abundance data in R Studio. To identify discriminative microbial features between strains, relative abundance data were analyzed using LDA effect size (LEfSe)(Segata et al. 2011), an algorithm developed for high-dimension data to identify features which characterize biological conditions. LEfSe was implemented through the Huttenhower Research Group Galaxy instance (http://huttenhower.sph.harvard.edu/galaxy/). Heritability was calculated as described by Hegmann and Possidente using the following formula $h2 = 0.5V_{\text{a}} /(0.5V_{\text{a}} + V_{\text{e}} )$ where V_a_ is the additive genetic variance and V_e_ is the average environmental variance (Hegmann and Possidente 1981).

### Evaluating relationships between 16s-based phylogeny, predicted function and metabolic phenotypes

Correlations between relative abundance of individual taxa and cardiometabolic phenotypes were assessed by spearman rho. All *p* values were adjusted using false discovery rate (FDR) of 10 % using the *q* value package (Storey 2002) in the R programing environment. To predict metagenomic functional content from our 16s rRNA survey, the software package Phylogenetic Investigation of Communities by Reconstruction of Unobserved States (Langille et al. 2013) (http://picrust.github.io/picrust/) was used. This computational approach exploits the relationship between phylogeny and function by combining 16s data with a database of reference genomes (GreenGenes) to predict presence of gene families. Briefly, OTUs were picked with a closed reference protocol against GreenGenes v 13.5 through QIIME. The resulting biom-formatted OTU table was uploaded to Galaxy (http://huttenhower.sph.harvard.edu/galaxy/) for 16s copy number normalization and metagenomic prediction. Functional predictions were exported as KEGG orthologs.

## Results

### Sequencing depth and paired-end stitching optimization

To determine the impact of stitching error stringency, and sequencing depth on global measures of community structure, and to fine-tune parameters for down-stream analysis, paired-end (2 × 250) Illumina MiSeq reads were stitched with varying levels of maximum permitted error (0, 1, 3, and 5 %) from mice fed the purified synthentic diet (AIN-93A). OTU tables resulting from the four levels of stitching error stringency were rarefied at multiple sequencing depths (1,000–96,000 reads/sample in steps of 5,000 reads/sample; 3 OTU tables generated per rarefaction point). This analysis highlighted a plateau in the number of taxa identified from a sequencing depth of approximately 50,000 reads/sample onward (Supplementary Fig. 1). Hence for all down-stream analyses, a rarefaction depth of 51,000 reads/sample was deemed acceptable. The numbers of taxa identified at each taxonomic ranking were comparable between stitching stringency levels, although a drop-off for maximum permitted error of 0 and 1 % was seen, likely due to the reduction in sample size which occurred with increased stringency at higher sequencing depths. Decreasing permitted stitching error increased Shannon Diversity Index, a measure of alpha-diversity, which is due at least in part to a decrease in evenness (as assessed using the GINI coefficient) as permitted error in stitching decreased (Supplementary Fig. 1). Based on these comparisons, down-stream analyses were conducted using a maximum permitted error of 3 %, and a sequencing depth of 51,000 reads/sample. These parameters provided the greatest compromise between maximizing diversity in the dataset and minimizing sample size drop-off, while permitting the lowest possible error rate during paired-end read stitching.

### Cardiometabolic phenotypes are differentially influenced by genetic background and atherogenic feeding

**Fig. 1:**
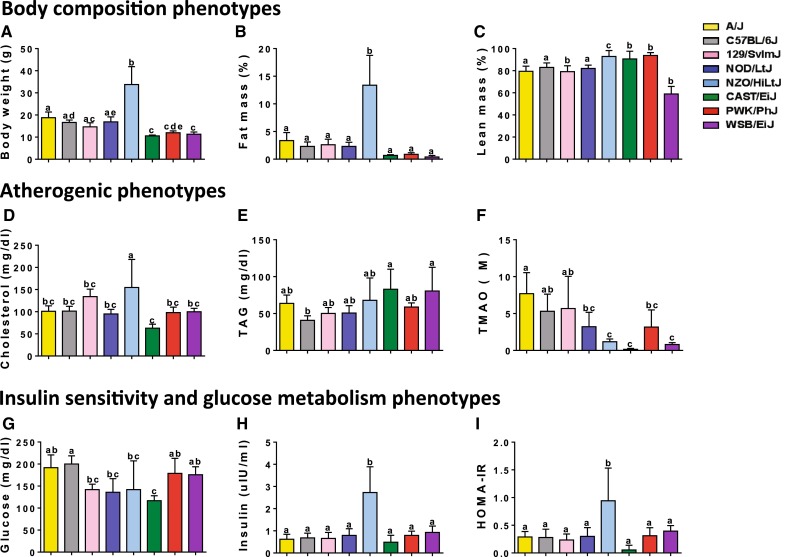
Cardiometabolic phenotypes between inbred mouse strains fed a synthetic diet. Inbred mouse strains were purchased from Jackson Laboratories at ~6 weeks, and placed on purified synthetic diet (AIN-93). After 2 weeks, animals were weight, body composition assessed by MRI and fasting plasma samples collected. Differences in bodyweight (**a**), and body composition by MRI are depicted as percentage fat mass (**b**), percentage of lean mass (**c**). Fasting plasma levels of cholesterol (**d**), triglycerides (**e**) and TMAO (**f**) were quantitated. Measures of insulin sensitivity were assessed and included plasma glucose (**g**), insulin (**h**) and calculated HOMA-IR (**i**). *p*-value for ANOVA <0.05 for all phenotypes. Significant between-strain differences identified with Tukey’s HSD post hoc test. Strains not sharing letter are significantly different (*p* < 0.05)

Mice are effective models of cardiovascular disease (Reardon and Getz 2001), and thus we quantitated these mice for the following traits associated with cardiovascular disease (Go et al. 2013) which we refer to as cardiometabolic traits: body weight, adiposity, and plasma levels of lipids, insulin, glucose and trimethylamine N-oxide (TMAO). Differences in cardiometabolic risk factors between strains of mice fed purified synthetic diet (AIN-93 M) are shown in Fig. 1. Significant differences were seen across strains for body weight (ANOVA *F* = 42.31, *p* < 0.0001), % lean mass (ANOVA *F* = 97.09, *p* < 0.0001), % fat mass (ANOVA *F* = 34.11, *p* < 0.0001), total cholesterol (ANOVA *F* = 9.7, *p* < 0.0001), plasma TAG (ANOVA *F* = 4.6, *p* < 0.0001), TMAO (ANOVA *F* = 9.12, *p* < 0.0001), fasting plasma glucose (ANOVA *F* = 7.09, *p* < 0.0001), fasting insulin (ANOVA *F* = 13.96, *p* < 0.0001), and HOMA-IR (ANOVA *F* = 7.5, *p* < 0.0001) (Fig. 1). These plasma lipid levels and adiposity for the inbred strains in our study are comparable to previously reported studies at the Mouse Phenome Database (www.phenome.jax.org) (Grubb et al. 2014). Body weight was comparable to Paigen 1; total cholesterol and TAG were comparable to Paigen 2, and adiposity measures were comparable to Naggert 1.

**Fig. 2:**
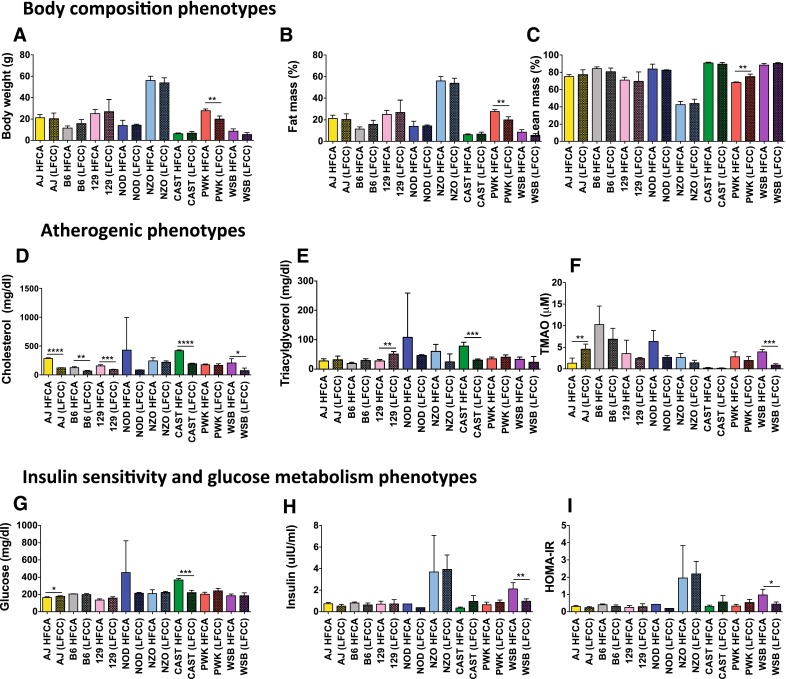
Atherogenic diets induce strain-dependent differences in cardiometabolic phenotypes. Following 16 weeks on either a high-fat cholic acid (HFCA; *open bars*) diet or low-fat cholesterol-containing diet without cholic acid (LFCC; *shaded bars*). Differences in Bodyweight (**a**), and body composition by MRI are depicted as percentage fat mass (**b**), percentage of lean mass (**c**). Fasting plasma levels of cholesterol (**d**), triglycerides (**e**) and TMAO (**f**) were quantitated. Measures of insulin sensitivity were assessed and included plasma glucose (**g**), insulin (**h**) and calculated HOMA-IR (**i**). Significant within-strain differences between diet groups assessed by independent t-tests. *p < 0.05, **p < 0.01, ***p < 0.001, ****p < 0.0001

Strain- and diet-dependent changes in cardiometabolic related traits (cholesterol, TAG, adiposity, insulin, and TMAO) were evident after 16 weeks of the diet treatment (Fig. 2), highlighting the influence of genetic background on diet-driven susceptibility to disease. A significant strain effect (*F* = 11.81, *p* < 0.0001) was seen for percentage fat mass change and glucose (*F* = 8.42, *p* < 0.0001) (Supplementary Fig. 2). Significant strain and diet effects (HFCA compared to LFCC) were seen for change in total plasma cholesterol concentrations (*F* = 2.56, *p* = 0.67; *F* = 7.34, *p* = 0.009, strain and diet, respectively). A significant strain (*F* = 8.62, *p* < 0.0001) and strain–diet interaction (*F* = 2.87, *p* = 0.017) was seen for plasma TMAO change from baseline (Supplementary Fig. 2). 2-way ANOVA of post-diet phenotypes revealed a significant independent effect of strain only on final body weight (*F* = 109.598, *p* < 0.0001), % fat mass (*F* = 94.54, *p* < 0.0001), % lean mass (*F* = 91.17, *p* < 0.0001), glucose (*F* = 3.60, *p* < 0.0038), insulin (*F* = 7.65, *p* < 0.0001), and HOMA (*F* = 7.53, p = 0.0001). A significant main effect of diet only was seen for total plasma fasting cholesterol (*F* = 8.85, *p* = 0.0047). Regulation of plasma TMAO levels is complex and is highly influenced by both the effect of genetic background of the mice (F = 12.97, *p* < 0.0001) and in response to dietary treatment (*F* = 6.32, *p* = 0.0159) (Fig. 2f). We observed a significant strain–diet interactions for plasma TMAO (*F* = 2.96, *p* 0.0129) (Supplemental Fig. 2E).

### Microbial community structure is driven by host genetic background

The baseline V4 amplicon library contained 5,490,587 reads after quality filtering (average 103,596/sample; range = 128–285,838 reads/sample). 3 samples were removed from the dataset as assigned reads fell below the rarefaction point of 51,000 reads/sample. The final sequence library comprised 42,130 assigned OTUs which collapsed to 82 bacterial taxa.

To explore the influence of genetic background on alpha-diversity under baseline nutrient intake, Shannon diversity index as well as the number of observed OTUs was calculated. This analysis revealed a significantly reduced diversity in PWK/PhJ samples compared with all other mouse strains (Supplementary Fig. 3). GINI coefficient, a measure of evenness in abundance distribution across OTUs within a sample, was modestly but significantly increased in PWK/PhJ samples, suggesting a more even distribution of abundances among bacterial taxa in the PWK/PhJ intestinal environment. Lower levels of alpha-diversity have been related to adverse metabolic phenotypes and reduced responsiveness to weight loss interventions (Cotillard et al. 2013; Le Chatelier et al. 2013).

**Fig. 3:**
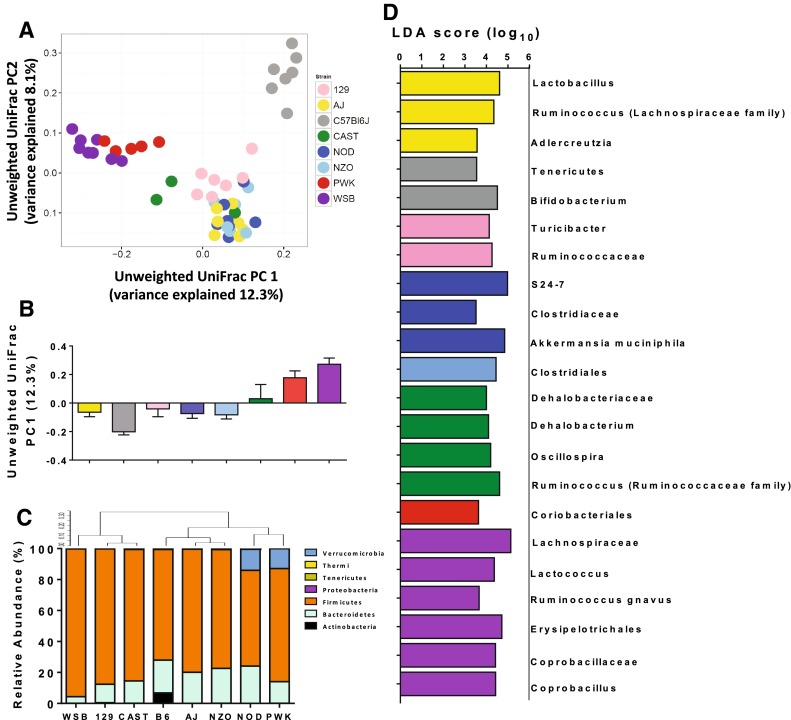
Global regulation of intestinal microbiome communities by genetic background in mice fed a purified synthetic diet. (**a**) Principle coordinates analysis (PCoA) of unweighted uniFrac. (**b**) Principle component 1 of unweighted UniFrac. (**c**) Phyla level relative abundance data. (**d**) Linear discriminant analysis with effect size (LEfSe) identified differentially abundant taxa between mouse strains. Taxa enriched in A/J (*yellow*), C57Bl6/J (*gray*), 129S1/SvlmJ (*pink*), NOD/ShiLtJ (*dark blue*), NZO/HiLtJ (*light blue*), CAST/EiJ (*green*), PWK/PhJ (*red*), and WSB/EiJ (*purple*) meeting LDA significant threshold >2 are shown

To determine the impact of strain on intestinal phylotypic diversity, UniFrac—a measure of phylogenetic similarity—was performed. Principle Coordinates Analysis (PCoA) of unweighted UniFrac distances revealed a strong effect of host genetics on gut microbial community membership. A distinct cluster consisting of C57BL/6J samples and a second cluster consisting of the wild-derived strains PWK/PhJ and WSB/EiJ were observed, with the remaining mouse strains clustering more closely in a third group (Fig. 3a and b). Analysis of Similarity (ANOSIM) on this UniFrac distance matrix revealed a significant difference between mouse strains (*r* = 0.868, *p* = 0.001). PERMANOVA confirmed a significant effect of mouse strain on microbial community structure (PERMANOVA *R* ^2^ = 0.33, *p* < 0.0001).

At a phylum level, the abundance of Firmicutes was dominant across inbred strains (range 61.8–95.4 %) (Fig. 3c), as has been described previously (Spor et al. 2011). Bacteroidetes was prominent in all samples (range 4.2–24.1 %). Gram-positive Actinobacteria were enriched in C57BL/6J samples only (6.8 % vs <0.2 % in all other samples), and the relatively recently described phylum, Verrucomicrobia, was enriched in PWK/PhJ and NOD/ShiLtJ only (12.9 and 14.1 % relative abundance respectively, vs <0.2 % in all other mouse strains). Other phyla detected at low abundance (<1 %) were Thermi, Tenericutes, and Proteobacteria. Together these phyla comprised 0.003–0.56 % of total community composition (Fig. 3c).

Linear Discriminant Analysis (LDA) was used to identify differentially abundant taxa between groups of inbred strains (Fig. 3d). Feces of C57Bl/6J animals was enriched for Actinobacteria (class) and Tenericutes (phylum) (Fig. 3d), and NOD/ShiLtJ samples were enriched for Verrucomicrobia, specifically *A. muciniphila*, the only member of the Verrucomicrobia phylum identified in this dataset, similar to that reported elsewhere (Derrien et al. 2011). The majority of discriminatory factors were members of the Firmicutes phylum, consistent with this being the dominant phylum across samples. Interestingly, wild-derived inbred strains (CAST/EiJ, PWK/PhJ, WSB/EiJ) are enriched for several genus-level members of Clostridiales, a dominant class of commensal bacteria (*Dehalobacterium*, *Rumminococcus*, *Oscillospira* genera), in addition to the genera *Lactococcus* and *Coprobacillus* (Firmicutes phylum) (Fig. 3d).

As genetics has previously been reported to exert greatest control at the tips of the phylogenetic tree (Benson et al. 2010), heritability estimates using genus level taxa relative abundances were calculated (Table 1). Our analysis further confirms both the influence of genetic background on microbial community structure and the specific nature of the regulation of individual taxa, as we identify a wide range of heritability for genus-level taxa (26–86 %).

**Table 1:** Heritability estimates of genus level taxa in CC/DO founder strains on AIN-93 diet (baseline group)

Genus Level Taxa	h2
Actinobacteria/Actinobacteria/Bifidobacteriales/Bifidobacteriaceae/Bifidobacterium	0.78
Bacteroidetes/Bacteroidia/Bacteroidales/Bacteroidaceae/Bacteroides	0.47
Bacteroidetes/Bacteroidia/Bacteroidales/Porphyromonadaceae/Parabacteroides	0.30
Firmicutes/Bacilli/Bacillales/Alicyclobacillaceae/Alicyclobacillus	0.22
Firmicutes/Bacilli/Bacillales/Bacillaceae/Bacillus	0.30
Firmicutes/Bacilli/Bacillales/Staphylococcaceae/Salinicoccus	0.30
Firmicutes/Bacilli/Bacillales/Staphylococcaceae/Staphylococcus	0.43
Firmicutes/Bacilli/Lactobacillales/Aerococcaceae/Abiotrophia	0.26
Firmicutes/Bacilli/Lactobacillales/Enterococcaceae/Enterococcus	0.64
Firmicutes/Bacilli/Lactobacillales/Lactobacillaceae/Lactobacillus	0.74
Firmicutes/Bacilli/Lactobacillales/Streptococcaceae/Lactococcus	0.69
Firmicutes/Bacilli/Lactobacillales/Streptococcaceae/Streptococcus	0.37
Firmicutes/Bacilli/Turicibacterales/Turicibacteraceae/Turicibacter	0.29
Firmicutes/Clostridia/Clostridiales/Dehalobacteriaceae/Dehalobacterium	0.73
Firmicutes/Clostridia/Clostridiales/Lachnospiraceae/Ruminococcus	0.61
Firmicutes/Clostridia/Clostridiales/Lachnospiraceae/Anaerostipes	0.30
Firmicutes/Clostridia/Clostridiales/Lachnospiraceae/Blautia	0.61
Firmicutes/Clostridia/Clostridiales/Lachnospiraceae/Butyrivibrio	0.59
Firmicutes/Clostridia/Clostridiales/Lachnospiraceae/Coprococcus	0.19
Firmicutes/Clostridia/Clostridiales/Lachnospiraceae/Dorea	0.28
Firmicutes/Clostridia/Clostridiales/Lachnospiraceae/Roseburia	0.84
Firmicutes/Clostridia/Clostridiales/Peptococcaceae/rc4/4	0.78
Firmicutes/Clostridia/Clostridiales/Ruminococcaceae/Anaerotruncus	0.82
Firmicutes/Clostridia/Clostridiales/Ruminococcaceae/Faecalibacterium	0.40
Firmicutes/Clostridia/Clostridiales/Ruminococcaceae/Oscillospira	0.76
Firmicutes/Clostridia/Clostridiales/Ruminococcaceae/Ruminococcus	0.69
Firmicutes/Clostridia/Coriobacteriales/Coriobacteriaceae/Adlercreutzia	0.50
Firmicutes/Erysipelotrichi/Erysipelotrichales/Coprobacillaceae/Coprobacillus	0.43
Firmicutes/Erysipelotrichi/Erysipelotrichales/Erysipelotrichaceae/Eubacterium	0.58
Firmicutes/Erysipelotrichi/Erysipelotrichales/Erysipelotrichaceae/Allobaculum	0.53
Proteobacteria/Alphaproteobacteria/Rhizobiales/Brucellaceae/Ochrobactrum	0.44
Proteobacteria/Alphaproteobacteria/Rhizobiales/Methylobacteriaceae/Methylobacterium	0.35
Proteobacteria/Alphaproteobacteria/Sphingomonadales/Sphingomonadaceae/Sphingomonas	0.26
Proteobacteria/Betaproteobacteria/Burkholderiales/Comamonadaceae/Tepidimonas	0.27
Proteobacteria/Deltaproteobacteria/Desulfovibrionales/Desulfovibrionaceae/Bilophila	0.32
Proteobacteria/Deltaproteobacteria/Desulfovibrionales/Desulfovibrionaceae/Desulfovibrio	0.57
Proteobacteria/Epsilonproteobacteria/Campylobacterales/Campylobacteraceae/Arcobacter	0.35
Proteobacteria/Gammaproteobacteria/Enterobacteriales/Enterobacteriaceae/Enterobacter	0.30
Proteobacteria/Gammaproteobacteria/Pasteurellales/Pasteurellaceae/Haemophilus	0.26
Proteobacteria/Gammaproteobacteria/Pseudomonadales/Moraxellaceae/Acinetobacter	0.41
Proteobacteria/Gammaproteobacteria/Pseudomonadales/Pseudomonadaceae/Pseudomonas	0.30
Tenericutes/Mollicutes/Anaeroplasmatales/Anaeroplasmataceae/Anaeroplasma	0.48
Verrucomicrobia/Verrucomicrobiae/Verrucomicrobiales/Verrucomicrobiaceae/Akkermansia	0.62

Whereas phylotyping has enabled an understanding of the taxonomic distribution and diversity of enteric microbial communities, understanding the functional metabolic underpinnings of the intestinal microbiota can provide significant depth to our understanding of microbiota in health and disease. Using PICRUSt, a computational approach to infer metagenome content from 16 s data, the metagenomic metabolism was predicted and seen to vary across strains, with significant between-strain differences seen in aspects of metabolism (amino acid, lipid, glycan, terpenoids and polyketoids, and secondary metabolites) (Supplementary Fig. 4), as well as pathways involved in basic functioning (cellular processes, environment, and genetic information processing). Significant differences in pathways enriched for genes involved in diseases (cancers, cardiovascular disease, immune system, infectious disease, metabolic disorders), and organ systems (digestive, endocrine) were observed (Supplementary Fig. 4), many of which were significantly related to cardiometabolic phenotypes (Supplementary Fig. 5).

### Atherogenic diet-driven shifts in microbial communities are strain-dependent

V4 16s rRNA libraries amplified from post 16 week diet intervention cecum DNA samples contained 6,388,210 reads after quality filtering (average 110,141/mouse; range = 298–212,914 reads/sample). 2 samples were removed from the dataset as assigned reads fell below the rarefaction point of 51,000 reads/sample. This sequence library comprised 29,326 assigned OTUs which also collapsed to 82 distinct bacterial taxa.

To investigate potential atherogenic diet-associated shifts in microbial community structure, several measures of alpha-diversity were conducted. Including all three diet groups in the analysis (AIN-93 M, HFCA, LFCC), 2-way ANOVA analysis revealed significant diet, strain, and diet–strain interactions on GINI co-efficient (*F* = 8.48, *p* < 0.0001; *F* = 72.773, *p* < 0.001; *F* = 3.698, *p* < 0.0001; diet, strain, and diet–strain, respectively), Shannon Diversity Index (*F* = 6.51, *p* < 0.0001; *F* = 122.4, *p* < 0.0001; *F* = 5.37; *p* < 0.0001 diet, strain, and diet–strain, respectively), and number o*F* observed OTUs (*F* = 13.46, *p* < 0.0001; *F* = 116.77, *p* < 0.0001; *F* = 5.50, *p* < 0.0001 diet, strain, and diet–strain, respectively). Compared with fecal AIN-93M samples, cecal samples from both HFCA and LFCC diet groups exhibited reduced alpha-diversity in a strain-dependent manner (Supplementary Fig. 6), with A/J, C57BL/6J, 129S1/SvlmJ, NOD/ShiLtJ, NZO/HILtJ, and WSB/EiJ experiencing a decrease in all measures of alpha-diversity from AIN-93M to HFCA (Supplementary Fig. 6). This reduction in alpha-diversity was less evident between the baseline and LFCC diet groups, with only A/J, and 129S1/SvlmJ experiencing a significant reduction. Consistent with the antimicrobial effects of cholic acid (Islam et al. 2011), alpha-diversity was reduced in HFCA samples compared with LFCC, although this reduction was significant for A/J, C57BL/6J, NOD/ShiLtJ, and NZO/HILtJ animals only (Supplementary Fig. 6).

**Fig. 4:**
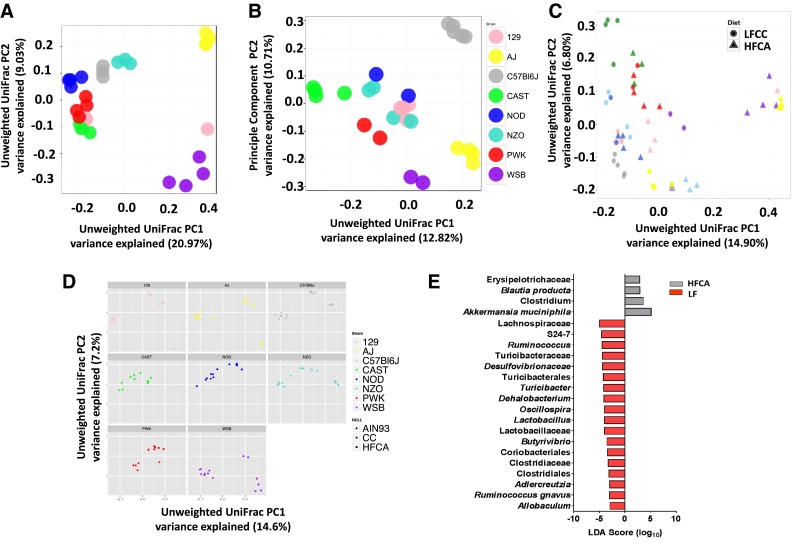
Global diet and strain-driven regulation of microbial beta-diversity. PCoA of Unweighted UniFrac of: (**a**) HFCA samples only; (**b**) LFCC samples only; (**c**) HFCA and LFCC samples combined; and, (**d**) HFCA, LFCC and AIN93M samples faceted by strain; (**e**) Linear discriminant analysis with Effect Size (LEfSe) identified differentially abundant taxa between HFCA and LFCC diet groups. Taxa enriched in HFCA diet (*gray*) and those enriched in LFCC diet (*red*) meeting LDA significant threshold >2 are shown. Taxa enriched in LF versus HFCA diet have a negative LDA score

When the HFCA and LFCC datasets were combined, a significant effect of strain remained evident (PERMANOVA *R* ^2^ = 0.31, *p* < 0.0001), and a significant effect of diet (PERMANOVA *R* ^2^ = 0.065, *p* < 0.0001) as well as a significant strain–diet interaction effect (PERMANOVA *R* ^2^ = 0.20, *p* < 0.0001) on beta-diversity were observed (Fig. 4C). However, analyzing the HFCA and LF diet groups in isolation revealed a more pronounced effect of strain on microbial community structure (HFCA group PERMANOVA for strain *R* ^2^ = 0.55, *p* < 0.0001; LF group PERMANOVA for strain *R* ^2^ = 0.54, *p* < 0.0001) (Fig. 4a and b), indicating that environmental challenges such as increased bile acids and dietary fat can lead to a sharper discrimination between strains, and highlighting the influence of genetic background in mediating the microbial response to diet. Furthermore, certain strains (A/J, C57BL/6J, WSB/EiJ) separated clearly by diet, while other potentially less dietary responsive strains (NOD/ShiLtJ, CAST/EiJ) cluster less distinctly (Fig. 4D).

Due to differences in sampling sites, direct comparisons between the baseline (feces) and post-diet (cecum) samples were not made. However, a high-level view of phyla differences between fecal microbial communities at baseline (AIN-93M), and cecal samples from both HFCA and LFCC diet groups show an increase in relative abundances of Verrucomicrobia and Bateroidetes with corresponding decreases in Firmicutes (data not shown) after 16-week dietary intervention. The increased abundance of the mucin-degrading Verrucomicrobia post diet may be driven by sampling site differences (feces at baseline and cecum post diet), as greater mucin production and also greater *A. muciniphila* (Verrucomicrobia phylum) has been reported in the cecum compared with other intestinal sites (Derrien et al. 2011).

**Fig. 5:**
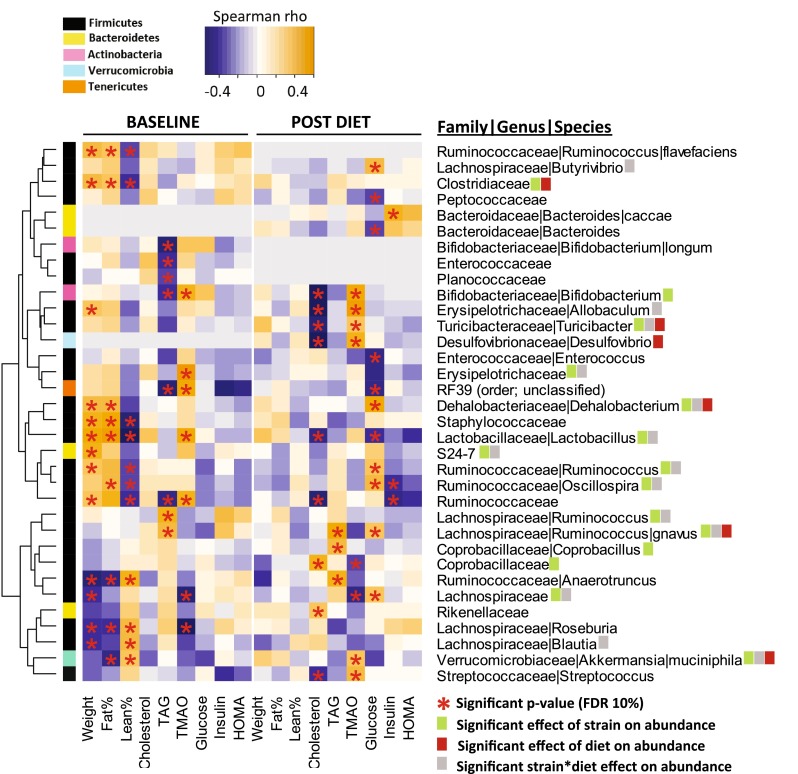
Significant relationships between microbial taxa and cardiometabolic phenotypes exist in mice fed purified synthetic diets (*baseline*) and atherogenic diets for 16 weeks (*post diet*). Correlations between taxa and phenotype assessed by Spearman rho. *Asterisks* denotes significant relationship (*p* values adjusted FDR 10 %)

To determine differentially abundant taxa between HFCA and LFCC diet groups, post diet relative abundance data were analyzed using LDA. This analysis identified 20 differentially abundant taxa (Fig. 4E) regulated by the two HFCA and LFCC cholesterol containing diets. In addition to a main effect of diet on these taxa, significant diet–strain interactions were observed for the genera *A. mucinipila* (phylum Verrucomicrobia), *Clostridium*, *Dehalobacterium*, *Rumminococcus gnavus*, *Turicibacter* (phylum Firmicutes), *Desulfovibrio* (phylum Proteobacteria), the order Coriobacteriales (phylum Firmicutes), and the families Clostridiaceae, Rumminococcaceae (phylum Firmicutes), and S2-47 (phylum Bacteroidetes). Many of these genetic and dietary regulated taxa form part of the core group of cardio-metabolic related taxa, described in detail below, related to body weight, body composition (proportions of lean and fat mass), plasma lipids, and the atherogenic metabolite TMAO (Fig. 5).

### Baseline and diet-induced relationships between microbial taxa and key cardiometabolic phenotypes

After correction for multiple testing, we identified significant relationships between phenotypes and a core group of cardiometabolic-related microbial taxa (Fig. 5).

On purified synthetic diet (AIN-93 M, baseline nutritional conditions), fasting plasma TMAO concentrations, a metabolite of microbial and dietary-derived TMA, was negatively correlated with butyrate producers such as unclassified microbiota in the Lachnospiraceae family (phylum Firmicutes, order Clostridiales), as well as the *Roseburia* genus within the Lacnospiraceae family. TMAO was positively correlated with the genus *Bifidobacterium* (phylum Actinobacteria), as well as unclassified members of the RF39 order (Tenericutes phylum, class Mollicutes) (Fig. 5). Positive relationships between TMAO and Rumminococcaceae and Erysipelotricaceae (families within Firmicutes phylum), and the genus *Lactobacillus* (Firmicutes phylum), were also identified (Fig. 5). Several of these relationships (*Bifidobacterium*, *Lactobacillus*, Erysipelotrichaceae) were maintained following atherogenic dietary intervention (Fig. 5), suggesting that these are stable genotype-driven taxa ~ phenotype relationships which are less influenced by the environmental effects of atherogenic dietary nutrients (cholesterol, fat, cholic acid). Following atherogenic diet consumption, several new relationships between TMAO and microbial taxa were identified. TMAO was positively correlated with *Turicibacter* and *Streptococcus*, two genera within class Bacilli (phylum Firmicutes), unclassified members of Peptostropoccaceae family (phylum Firmicutes), as well as the genus *Desulfovibrio* (phylum Proteobacteria). A diet-induced negative relationship was identified between TMAO and Coprobacillaeae (phylum Firmicutes).

At baseline, significant relationships were identified between body weight and body composition (fat and lean %) (Fig. 5). Several genera within the Clostridiales order (*Anaerotruncus*, *Blautia*, *Roseburia*) exhibited opposing relationships between weight/fat and lean mass (Fig. 5). However, although the direction of many of these relationships was retained post diet, the magnitude decreased, and none of these baseline relationships reached significance post diet. Additionally, no novel atherogenic diet-induced relationships were identified for these body composition phenotypes.

At baseline, fasting plasma TAG concentrations were negatively correlated with relative abundance of unclassified members of the order RF39 (phylum Tenericutes, class Mollicutes), and families Planococcoceae, Enterococcaceae), and Ruminocoocoeae (all Firmicutes phylum). TAG was positively correlated with relative abundance of *R. gnavus* (family Lachnospiraceae, phylum Firmicutes) (Fig. 5). This positive relationship with *R. gnavus* persisted following atherogenic dietary intervention (Fig. 5). Dietary intervention increased the strength of the positive relationship between TAG and the genera *Anaerotruncus* and *Coprobacillus* (phylum Firmicutes), and these relationships reached significance at the post-diet time-point only (Fig. 5). Other relationships between plasma TAG and microbial taxa abundance were lost after atherogenic feeding (Fig. 5).

Although no significant relationships existed between plasma total cholesterol and taxa at baseline, after atherogenic feeding, diet-induced positive relationships between fasting plasma cholesterol and family Rikenellaceae (phylum Bacteroidetes), and Coprobacillaceae (phylum Firmicutes) were revealed (Fig. 5). Additionally, dietary-responsive negative relationships between genera *Bifidobacterium* (phylum Actinobacteria), *Allobaculum*, *Turicibacter*, *Lactobacillus*, *Streptococcus* (phylum Firmicutes), and *Desulfovibrio* (phylum Proteobacteria) were identified (Fig. 5).

No significant correlations were identified between glucose, insulin or HOMA-IR and microbial abundance at baseline, however, at the post diet time-point, fasting plasma glucose was negatively correlated with genera *Lactobacillus*, *Enterococcus* (class Bacilli, phylum Firmicutes), and families Peptococcaceae and Erysipelotrichaceae (phylum Firmicutes). Glucose was positively correlated with genera *Ruminococcus* and *Oscillospira* (family Rumminococcaceae), and *Dehalobacterium* (family Dehalobacteriaceae), *Butyribrio* and *R. gnavus* (family Lachnospiraceae) (all Clostridiales order, phylum Firmicutes). Atherogenic feeding strengthened the negative relationship between Rumminococcaeae and insulin, reaching significance post diet only, and revealed a novel positive dietary-responsive relationship between *Bacteroides caccae* (phylum Bacteroidetes) and insulin (Fig. 5).

## Discussion

Recent reports have highlighted interactions between the microbiome and metabolism of dietary components such as phosphatidylcholine and carnitine on modulating cardiovascular disease risk (Koeth et al. 2013a; Tang et al. 2013; Wang et al. 2011), and have identified numerous associations between the microbiome and metabolic diseases. In the current report, we use a segregating panel of inbred mouse strains, representing ~90 % of the genetic variation in mice (Roberts et al. 2007), to examine the effects of genetics on intestinal microbiota and cardiometabolic risk factor response to diet. There are five main findings of our current studies: (1) differences in enteric microbial communities between inbred mouse strains are evident; (2) some of these strain-driven differences are retained and become more pronounced with dietary intervention; (3) microbial and phenotypic response to diet varies by mouse strain; (4) differences between the mouse strains suggest underlying differences in intestinal barrier function, inflammatory environment, short chain fatty acid (SCFA) production, and bile acid metabolism; and (5) the observed differences in a core group of microbial taxa are significantly related to cardiometabolic phenotypes. These results highlight not only the influence of genetic background on microbiome community structure but also on the microbial and phenotypic response to diet, and illustrate the effectiveness of the CC/DO founder strains in nutrigenomic studies.

The significant effect of mouse strain echoes that of recent work demonstrating that microbial composition in the intestine is a complex, polygenic trait. Numerous genetic studies in mice, including those using genetic reference panels (Benson et al. 2010; Campbell et al. 2012; Hildebrand et al. 2013; McKnite et al. 2012), have demonstrated an effect of genetic background on microbial diversity. Interestingly, Benson and co-workers demonstrated that multiple taxa can co-localize to a single QTL, suggesting that a single-genetic locus may regulate the abundance of several taxa (Benson et al. 2010). A previous survey using founder strains of the CC (Campbell et al. 2012) also reports a significant effect of mouse strain on microbial diversity, although few discriminating OTUs were identified, and differences were not linked to phenotype. Our current study has sharper discrimination between inbred mouse strains potentially due to ~10 fold increase in sequencing depth, differing sites of microbial assessment for the baseline comparison (cecum vs feces), and the use of a synthetic defined diet which avoid the variation inherent in non-purified diets (Reeves et al. 1993). In the current study, C57Bl6/J animals had differentially abundant members of the Actinobacteria phylum. These results mirror those of previous studies which have demonstrated an effect of the C57Bl6/J allele in a QTL regulating Actinobacteria abundance (Benson et al. 2010).

To ensure the host-microbial relationship remains harmonious, a sophisticated host antigen recognition and defense system has developed to prevent microbial invasion. Considering this tension between microbial populations and the host immune system, it is not surprising that immune function is a recurring theme in the host-genetic regulation of microbial abundance. A large proportion of QTL regions reported to regulate microbial abundance contain genes related to immunity and maintenance of barrier function (Benson et al. 2010; McKnite et al. 2012; Srinivas et al. 2013). The influence of immune-related genes is further evidenced by the dramatic effects on microbial community structure caused by mutations in single genes related to host immunity (Thompson et al. 2010; Wen et al. 2008). Hildebrand and co-workers show that microbiota cluster by fecal calprotectin concentrations, with high fecal calprotectin indicative of an inflammatory colonic environment (Hildebrand et al. 2013). Several taxa identified as being differentially abundant between strains in our study, such as members of the Clostridiales order, are known to be decreased in intestinal inflammatory environments (Schwab et al. 2014), suggesting that differences in immune response may underlie strain-dependent differences observed, as has been indicated by previous studies (Benson et al. 2010; McKnite et al. 2012; Srinivas et al. 2013).

In order to test genetic background, environmental forces must be steady and carefully controlled. Factors such as the maternal environment, litter effects, cage mates, the location that the mice are housed, and the commercial vendor, can influence microbial populations (Benson et al. 2010; Campbell et al. 2012; Friswell et al. 2010; Hildebrand et al. 2013). Currently, the relative strength of environmental versus genetic signals on microbial regulation is unclear. Uterine implantation studies have shown that mice of different genetic backgrounds have similar microbial composition when reared by the same foster mother, indicating that in certain circumstances, environmental drivers can overpower genetic influences at least for non-adherent bacterial populations (Friswell et al. 2010). As animals were purchased from a commercial vendor, the current study does not permit a full investigation of environmental forces influencing enteric bacterial populations. Although the existence of within strain cage effects has been clearly illustrated in the literature (McCafferty et al. 2013), these were not evident in the current study. Co-housing within strains during transportation, use of synthetic diet (as oppose to standard chow or non-purified diet which can be variable in composition), or singly housing for 3-days prior to fecal collection (during which time the impact of cage may have begun to rescind), are possible reasons for the close clustering seen within strains.

Considering that the microbiome depends on dietary and host-derived nutrients for survival, it is unsurprising that dietary intake has such a profound impact on the microbiome (Cotillard et al. 2013; Spor et al. 2011). Altered dietary nutrient composition can lead to a bloom or wane in certain microbiota with varying capacity to flourish under changing environmental conditions. Shifts in underlying microbial function and metabolism can have secondary effects on host metabolism. Rather than overwhelm the effect of mouse strain, dietary intervention resulted in a stronger main effect of mouse strain on microbial community structure. Interestingly, the separation within mouse strains by diet was variable across the inbred lines, and certain cardiometabolic-related taxa exhibited diet–genotype interaction effects, suggesting variable microbial responsiveness to external dietary influences as is seen phenotypically with these strains. Although this is a relatively new area of focus, other groups using mouse genetic reference populations (Parks et al. 2013b), or single gene knockout models (Kashyap et al. 2013) have demonstrated an interaction between microbiota and diet that is influenced by host genotype. Recent work with human participants reported that despite retained variation in taxonomy following dietary intervention, microbial gene expression, as assessed by RNAseq, clustered by diet group and exhibited less between-subject variation than at baseline (David et al. 2014). Considering this, it is unclear whether the taxonomic separation seen between strains post diet reflects variable metabolic functionality, which would in turn impact host phenotype. However, our core group of cardiometabolic-related taxa are good candidates for future focus.

The microbiome is a metabolically active, complex organ, producing many metabolites which can directly influence host phenotype. Genetic and dietary regulated taxa identified in this study were enriched for known SCFA, bile acid and inflammation and barrier function-related taxa. Recent efforts have focused on the link between the microbiome and the atherogenic metabolite TMAO. TMAO is formed from trimethylamine (TMA) via hepatic flavin mono-oxygenase 3 (FMO3) (Bennett et al. 2013a). The microbiome plays an obligate role in the formation of TMA (from trimethylamine-containing nutrients choline and carnitine), and antibiotic knockdown studies show clearly that TMAO is not formed in the absence of the microbiome (Tang et al. 2013). Bacterial species harboring putative choline utilization gene clusters (*cut*-*c*) have been suggested to play a central role in enteric TMA formation (Craciun and Balskus 2012) (and therefore down-stream TMAO production), however the specific microbiota have not been fully ascertained. In addition to the microbial differences in TMA production, differences in Fmo3 gene expression can contribute to TMAO levels as we have previously reported that *Fmo3* both has a cis-eQTL and is correlated with plasma TMAO levels (Bennett et al. 2013b). Thus, variation in the microbiome and *Fmo3* gene expression contribute to plasma TMAO levels. Our current studies focus on the genetic regulation of the gut microbiome and relationships with TMAO and other cardiovascular risk factors. A previous report that plasma TMAO and members of the Tenericutes phylum (Koeth et al. 2013b) are correlated was confirmed in our current study. Additionally, we see a significant negative correlation between plasma TMAO concentrations and members of the Lachnospiraceae family including the *Roseburia* genus, a known butyrate producer. Other members of this family were also negatively correlated although failed to reach significance after correction for multiple testing. We identified a number of dietary responsive relationships related to plasma TMAO levels, including positive relationships between the genus *Desulfovibrio*, a member of the sulfate/sulfite reducing Desulfovibrionaceae family. A species within the *Desulfovibrio* genus has been demonstrated to degrade choline to TMA (Craciun and Balskus 2012), hence an increased formation of this TMAO precursor may explain this positive relationship.

Several elegant studies have clearly established a role for the microbiome in regulation of body weight and adiposity (Backhed et al. 2004; Ridaura et al. 2013). A shared genetic regulation has also been reported, with genetic loci regulating body composition complex traits coinciding with loci regulating microbial abundances (McKnite et al. 2012; Parks et al. 2013a). The negative relationship between body weight and fat mass, and the positive relationship between lean mass and *Roseburia*, *Blautia* and other unclassified genera of the Lachnospiraceae family suggests a relationship between butyrate production in the intestine and body weight control. In addition to providing a nutrient source for the enteric epithelium (De Vadder et al. 2014), butyrate plays a role in modulating host energy expenditure (Gao et al. 2009). Additionally, butyrate can influence gut peptide secretion with potential secondary effects on satiety (Hosseini et al. 2011) via SCFA receptors such as GPR43 (Kimura et al. 2013).

The influences of bacterial taxa with opposing roles in barrier function on plasma lipids and other metabolic markers highlights the importance of barrier function for metabolic health. *Bifidobacterium longum*, known to promote tight junction integrity, was associated with decreased plasma TAG suggesting a protective role. On the other hand, *R. gnavus*, a mucin-degrading species associated with reduced barrier integrity and bile acid metabolism, was positively related to TAG and plasma glucose. Barrier function is crucial to guard against bacterial translocation and metabolic endotoxemia which has been directly linked to metabolic disease increased fasting glycemia, decreased glucose tolerance, increased body and liver weight, increase liver triglyceride content and increased energy intakes (Cani et al. 2007).

The genetic variation of the CC/DO founders coupled with dietary perturbation revealed a core group of genetically and dietary regulated microbial taxa, many of which are connected to cardiometabolic phenotype. Many of the taxa identified have strong plausible roles in mediating host phenotype, and as they appear regulated by both genetics and diet, may represent useful targets in understanding diet-induced variability in metabolic disease risk. The specific relationship of these taxa to human disease remains to be confirmed as the microbial composition of humans and mice are similar at the phyla level but may not contain the same specific species of microbiota (Ley et al. 2005).

In conclusion, our results provide a strong indication that host genetics drives microbial diversity under baseline nutrient conditions, and in response to atherogenic nutrient intake. We also highlight that genetic–diet influences on the microbiome are related to cardiometabolic phenotypes, suggesting that differences at the level of the intestinal microbiome may underlie some of the differences in mouse-strain dependent susceptibility to cardiovascular disease and metabolic ill health in response to atherogenic diets. Understanding how diet responsive microbiota are influenced by genetics and importantly contribute to host phenotype will be pivotal in fully realizing the potential for personalized nutrition and our comprehension of inter-individual variation in disease risk. Segregating panels of mice such as the CC/DO founders provide wide scope of variation which can be probed in depth in future studies. Underlying differences in gene expression and fecal metabolites will shed more light on host-derived changes in functional and metabolic capacity of the microbiome, and provide greater insight into mechanisms by which these changes influence host phenotype.

## Electronic supplementary material

Below is the link to the electronic supplementary material.

Supplementary material 1 (PDF 941 kb)
