# Supplementary material for: Responsiveness of cardiometabolic-related microbiota to diet is influenced by host genetics
Source: Mamm Genome. 2014 Aug 27;25(11):583–99. doi: 10.1007/s00335-014-9540-0 (PMC4239785; doi:10.1007/s00335-014-9540-0)
Supplement: Supplementary file 1 — Supplementary material 1 (PDF 941 kb) [file 335_2014_9540_MOESM1_ESM.pdf]

Data Supplement

Responsiveness of cardiometabolic-related microbiota to diet is influenced by host genetics

Annalouise O'Connor[1], Pamela M. Quizon[1], Jody E. Albright[1], Fred T Lin[1], Brian J. Bennett[1,2,3]

1. UNC Chapel Hill Nutrition Research Institute, 500 Laureate Way, Kannapolis, NC 28081
2. Department of Genetics, University of North Carolina Chapel Hill, NC 27599
3. Department of Nutrition, University of North Carolina Chapel Hill, NC 27599

CORRESPONDING AUTHOR: Brian J. Bennett, UNC Chapel Hill Nutrition Research Institute, 500 Laureate Way, Kannapolis, NC 28081. Email: bennettb@email.unc.edu Tel: (704) 250-5044

KEY WORDS: collaborative cross, nutrigenomics, atherogenic diet, microbiome, inbred mouse strains

RUNNING TITLE: Host genetics influences microbial response to diet

Supplementary Figure 1

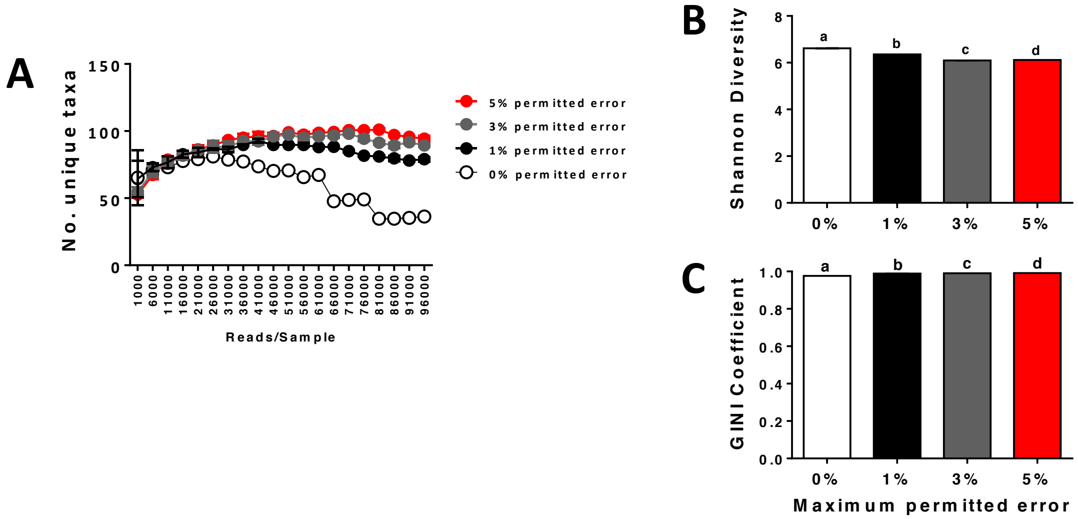

Supplementary Figure 1: Sequencing depth and stitching stringency influence microbial diversity

Supplementary Figure 2

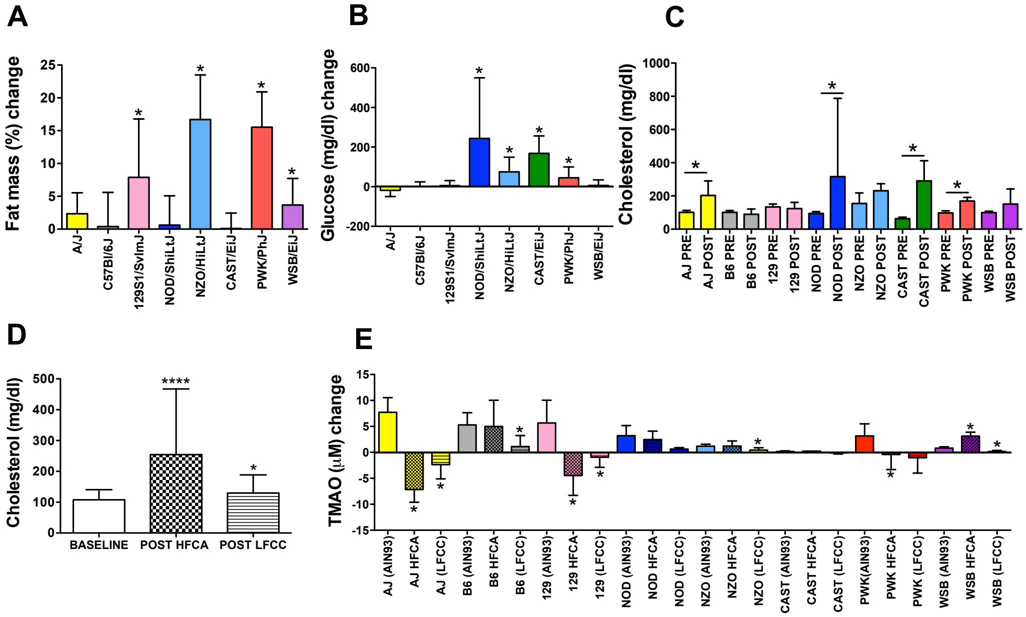

Supplementary Figure 2: Change in cardiometabolic phenotypes in response to diet varies by strain. Percent change in fat mass (A), and plasma glucose (B) following 16 weeks of the cholesterol-containing control diets (HFCA and LFCC) are shown for each strain in the study. Plasma cholesterol (C) levels are shown at baseline (mice fed AIN-93A) and following 16 weeks of the cholesterol-containing control diets (HFCA and LFCC). There was a significant effect of diet on plasma cholesterol levels (D). Percent change in plasma TMAO following 16 weeks of the cholesterol-containing control diets (HFCA and LFCC) are shown for individual diets.

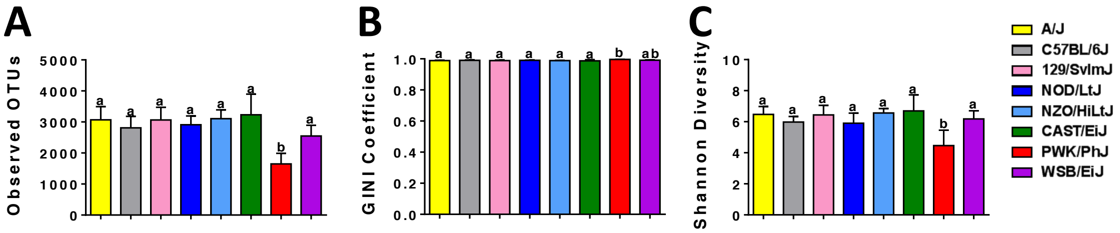

Supplementary Figure 3: Measures of alpha-diversity differ across inbred mouse strains. P-value for ANOVA < 0.05 for all phenotypes. Significant between-strain differences identified with Tukey's post-hoc test. Strains not sharing letter are significantly different ($p < 0.05$).

Supplementary Figure 4

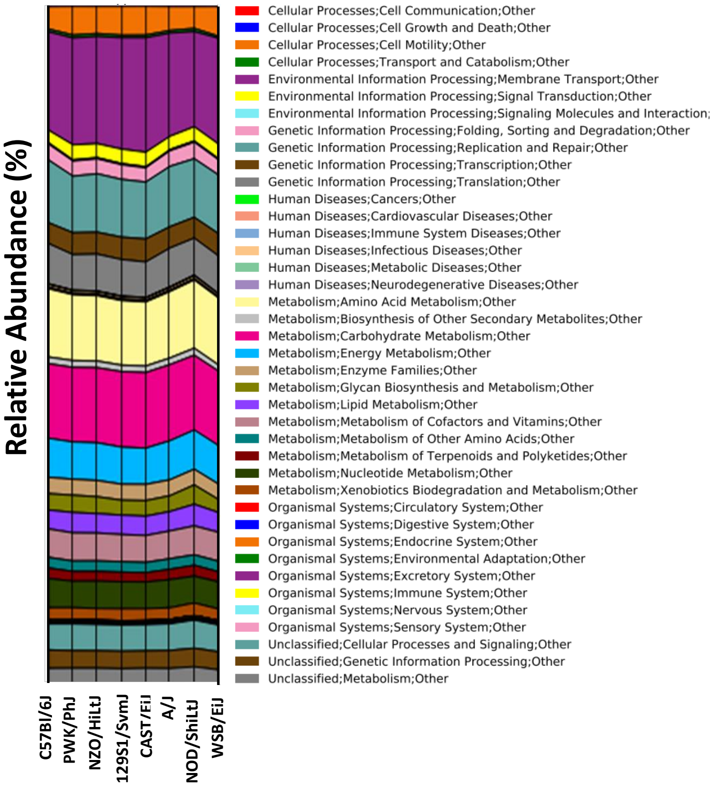

Supplementary Figure 4: Inferred metabolic function varies across inbred mouse strains. KEGG pathway gene abundance predicted from 16s rRNA sequence data using PICRUST. Between-strain differences assessed by ANOVA. * p <0.05, ** p<0.01, *** p<0.001, **** p<0.0001.

Supplementary Figure 5

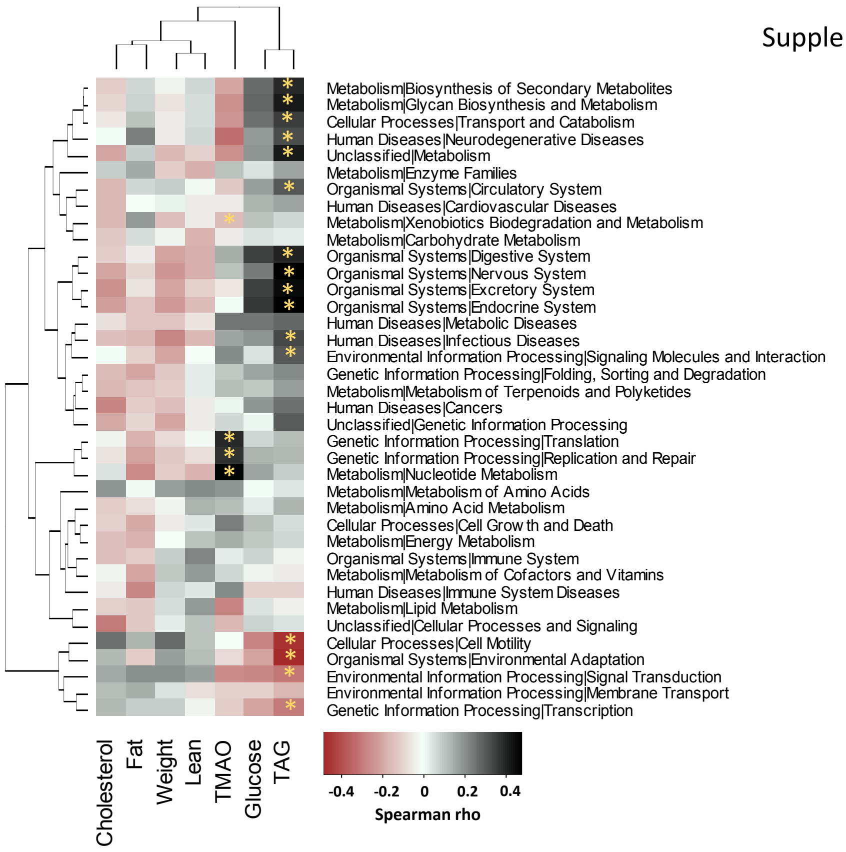

Supplementary Figure 5: Relationships between inferred KEGG abundance and cardiometabolic phenotype. Correlations between KEGG and phenotype assessed by Spearman rho. * denotes significant FDR (10%) adjusted p-value.

Supplementary Figure 6

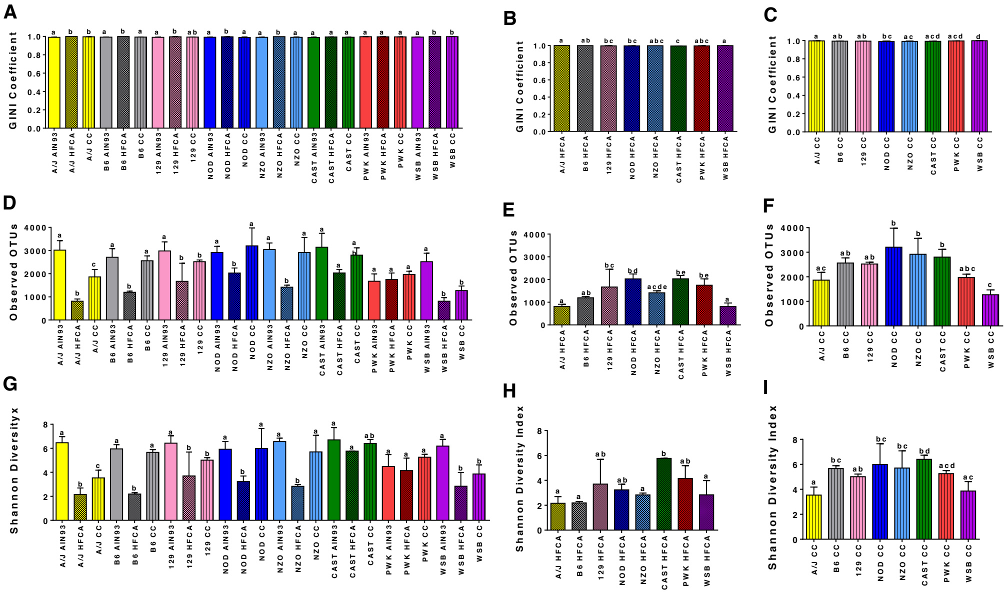

Supplementary Figure 6: Alpha diversity under baseline nutrient conditions and post 16 week post atherogenic diet intervention. Within strain diet-induced differences in alpha-diversity measures (A, D, G) were assessed by ANOVA with Tukey-HSD post-hoc test. Diets not sharing letters are significantly different ($p < 0.05$). Between strain HFCA-induced differences (B, E, H) were assessed by ANOVA. Strains not sharing letters are significantly different ($p < 0.05$). Between-strain CON-induced differences (C, F, I) were assessed by ANOVA. Strains not sharing letters are significantly different ($p < 0.05$).
